# A Novel Type II Photoinitiator with Self-Supplied Hydrogen for Anti-Creep Crosslinking Polyethylene Film

**DOI:** 10.3390/ma18061313

**Published:** 2025-03-16

**Authors:** Fei Yang, Zhaoyuan Jing, Yingqiu Wang, Guodong Jiang

**Affiliations:** College of Materials Science and Engineering, Nanjing Tech University, Nanjing 211816, China

**Keywords:** macromolecular photoinitiator, benzophenone (BP), molar extinction coefficient, UV crosslinking

## Abstract

Two macromolecular photoinitiators, bis(4-benzoylphenyl) malonate (BPMD) and bis(4-benzoylphenyl) 3,3’-(piperazine-1,4-diyl)bis(3-oxopropanoate) (DBPMD), were successfully synthesized from 4-hydroxybenzophenone (4-BP), malonyl chloride, and anhydrous piperazine. Structural characterization using Fourier transform infrared spectroscopy (FTIR) and proton nuclear magnetic resonance spectroscopy (^1^H NMR) confirmed the expected molecular framework. Ultraviolet (UV) absorption spectroscopy revealed that BPMD and DBPMD exhibited enhanced molar extinction coefficients and red-shifted absorption maxima compared to 4-BP. Migration studies in high-density polyethylene (HDPE) demonstrated significantly lower diffusion rates for BPMD and DBPMD than for 4-BP, with DBPMD exhibiting superior photoinitiation efficiency even in the absence of amine-based activators. Photoinitiation performance, photocrosslinking kinetics, and mechanical evaluations indicated that both BPMD and DBPMD enabled efficient UV-initiated crosslinking, leading to improved tensile strength and creep resistance in polyethylene films. These findings highlight the potential of BPMD and DBPMD as advanced photoinitiators for high-performance UV-crosslinked polyethylene systems.

## 1. Introduction

Polyethylene is one of the three major synthetic polymer materials, widely recognized for its lightweight nature, excellent chemical and water resistance, non-toxicity, environmental friendliness, and ease of processing and molding. Additionally, polyethylene exhibits superior electrical insulation, good toughness, impact resistance, wear resistance, and remarkable resistance to environmental stress cracking, making it highly versatile across various application domains [1,2,3]. However, due to its carbon-chain molecular structure with low polarity, the intermolecular van der Waals forces within polyethylene are relatively weak. As a result, polyethylene materials are highly susceptible to creep deformation, which significantly limits their applications in critical fields such as marine engineering, aerospace, military technology, medical devices, and construction [4,5,6]. 

To address these limitations, researchers have developed various crosslinking strategies, such as irradiation, chemical and UV light crosslinking [7,8,9]. Among these, UV crosslinking has garnered significant attention due to its advantages, including rapid processing speeds, low production costs, minimal energy consumption and the absence of pre-crosslinking requirements [10,11]. This technique facilitates polymerization reactions within a short time frame, making it a cost-effective and energy-efficient approach. 

UV crosslinking in polyethylene is initiated through the addition of UV photoinitiators during material mixing [12]. Upon UV irradiation, these photoinitiators absorb energy, transition to an excited state and subsequently generate alkyl radicals by abstracting hydrogen atoms from polyethylene [13,14]. These radicals propagate chain crosslinking, highlighting the critical role of photoinitiators in the crosslinking process [15]. Although photoinitiators are used in small quantities, their composition and concentration significantly influence the crosslinking rate and efficiency [16]. 

Traditional small-molecule photoinitiators, such as BP, face challenges related to their high mobility and volatility [17]. Their low molecular weight and short chains hinder entanglement with polyethylene molecules, leading to surface migration, uneven distribution and reduced internal crosslinking efficiency [18,19]. Moreover, under UV exposure, small-molecule photoinitiators can degrade into volatile organic compounds (VOC), which are toxic and detrimental to both human health and the environment [20]. Consequently, the development of photoinitiators with low volatility and migration is imperative for safe and efficient UV crosslinking. 

Macromolecular photoinitiators address these limitations by combining high molecular weight and extended chain lengths, reducing their volatility and migration [19,21]. Additionally, macromolecular photoinitiators such as benzophenone-based BPMD and DBPMD exhibit enhanced initiation efficiency without requiring co-initiators like active amines [22,23]. 

In this study, two benzophenone-based macromolecular photoinitiators, BPMD and DBPMD, were synthesized and characterized. DBPMD, containing active amine groups, was structurally verified using FTIR and ^1^H NMR. UV absorption properties and photodegradation behavior under methanol UV exposure were analyzed using UV spectrophotometer. Migration tendencies were assessed by immersing polyethylene films containing these photoinitiators in methanol and comparing them with traditional small-molecule initiators. Crosslinking performance was evaluated through gel content measurements under varying photoinitiator concentrations, light distances and crosslinker types [24]. The findings demonstrate the superior efficiency and safety of BPMD and DBPMD in polyethylene UV crosslinking applications. 

## 2. Experiment

### 2.1. Materials and Instruments

The materials used include 4-hydroxybenzophenone (4-BP, ≥98%) from Shanghai Merrell Test Equipment Co., Ltd. (Shanghai, China); benzophenone (BP, ≥98%) from Shanghai Anaiji Chemical Co., Ltd. (Shanghai, China); malonyl chloride (AR) from Shanghai Maclean’s Biochemical Technology Co., Ltd. (Shanghai, China); trimethylol- propane triacrylate (TMPTA, AR) from Shanghai Aladdin Biochemical Technology Co., Ltd. (Shanghai, China); anhydrous ethanol from Wuxi Yasheng Chemical Co., Ltd. (Wuxi, China); triethylamine (AR), methylene chloride (AR) and xylene (AR) from Shanghai Lingfeng Chemical Reagent Co., Ltd. (Shanghai, China).

The Instruments used include an electronic balance (ME104E/02) from Mettler Toledo Instruments Co., Ltd. (Shanghai, China); a circulating water vacuum pump (SHZ-D(III)) from Hangzhou Jingfei Instrument Technology Co., Ltd. (Hangzhou, China); a thermostat electric heating jacket (DZTW 500 mL) from Shanghai Lichen Bangxi Instrument Technology Co., Ltd. (Shanghai, China); and an electric constant-temperature drying oven (101-2B) from Shanghai Shangpu Instrument Equipment Co., Ltd. (Shanghai, China).

### 2.2. Synthesis of BPMD

A total of 23.78 g (0.12 mol) of 4-hydroxybenzophenone, 12.12 g (0.12 mol) of triethylamine and 150 mL of dichloromethane were added to a 500 mL three-necked flask and stirred under ice-water bath conditions for 20 min. Separately, 7.05 g (0.05 mol) of malonyl chloride and 100 mL of dichloromethane were prepared in a constant-pressure dropping funnel. The mixture was added dropwise to the reaction flask while maintaining the reaction temperature below 3 °C. After the addition, the reaction was allowed to proceed for 6 h.

**Figure 1:**
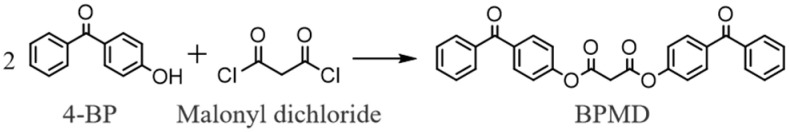
Synthetic route of BPMD.

Upon completion, the product was filtered, washed and recrystallized to yield the crude product. Further purification by column chromatography produced a white powdery sample of BPMD with a yield of 81.4%. The synthesis pathway is illustrated in Figure 1.

### 2.3. Synthesis of DBPMD

First, 15.51 g (0.11 mol) of malonyl chloride and 100 mL of dichloromethane were added to a 500 mL three-necked flask and stirred in an ice-water bath for 20 min. A solution containing 4.31 g (0.05 mol) of anhydrous piperazine, 12.12 g (0.12 mol) of triethylamine and 100 mL of methylene chloride was prepared separately and added dropwise over 100 min using a constant-pressure dropping funnel while maintaining the temperature below 3 °C. The reaction was allowed to proceed for 4 h after the addition. 

**Figure 2:**
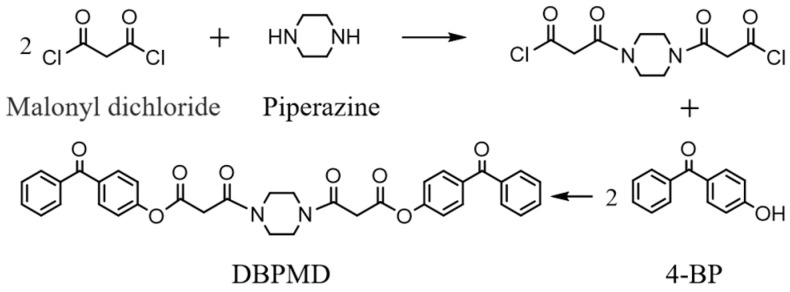
Synthetic route of DBPMD.

Subsequently, 23.78 g (0.12 mol) of 4-hydroxybenzophenone, 12.12 g of triethylamine and 100 mL of dichloromethane were mixed and added dropwise over 100 min, ensuring the temperature did not exceed 3 °C. After 6 h of reaction, the crude product was filtered, washed and recrystallized. Further column chromatography yielded a white powdery sample of DBPMD with a yield of 65.8%. The synthesis route is presented in Figure 2. 

### 2.4. FTIR Spectroscopy

The structures of BPMD and DBPMD were analyzed using FTIR spectrometer (Nicolet is5, Thermo Fisher Scientific Co., Ltd., Waltham, MA, USA). By observing characteristic peak shifts and intensity changes in the infrared spectra, the synthesis of the desired products was confirmed. The spectral range was set from 4000 to 500 cm^−1^ with a resolution of 0.5 cm^−1^. 

### 2.5. NMR Hydrogen Spectroscopy

NMR(ADVANCE III, Bruker Co., Ltd., Billerica, MA, USA) spectroscopy was employed to characterize the hydrogen resonance spectra of BPMD and DBPMD. Tetramethylsilane (TMS) served as the internal standard and deuterated chloroform (CDCl_3_) was used as the solvent.

### 2.6. Mass Spectrometry

Mass spectrometric(UltiMate 3000, ThermoFisher Co., Ltd., Walthan, MA, USA) characterization of BPMD and DBPMD was performed using methanol as the solvent. 

### 2.7. Elemental Analysis

Elemental analysis was performed to characterize BPMD and DBPMD using a elemental analyzer(UNICUBE, Elementar Analysensysteme GmbH Co., Ltd., Hanau, German). 

### 2.8. Differential Scanning Calorimetry (DSC)

The thermal properties of BPMD and DBPMD were analyzed using a DSC instrument (Q200, TA Instruments Co., Ltd., New Castle, DE, USA). Approximately 5–10 mg of sample was placed under a nitrogen atmosphere. The heating protocol involved increasing the temperature from 25 °C to 250 °C at a rate of 20 °C/min, holding for 5 min to erase thermal history, cooling to 25 °C at the same rate, holding for 5 min and reheating to 250 °C at 20 °C/min. 

### 2.9. UV Absorption Behavior

UV absorption spectrometry (UV-1100, MAPADA Co., Ltd., Shanghai, China) was used to measure the UV absorption spectra of BPMD and DBPMD. A solution containing the photoinitiator with a benzophenone group at a concentration of 5 × 10^−^⁵ mol/L was prepared in methanol. The absorption spectrum was recorded over the range of 200–350 nm to identify the main absorption regions, the maximum absorption wavelength and the maximum molar extinction coefficient. The molar extinction coefficient was calculated using the following Equation (1) [19]:

(1) $$\varepsilon =\frac{A}{c\cdot l}$$

where ε is the molar extinction coefficient; A is the absorbance; c is the concentration of photoinitiator, mol/L; l is the optical path length (liquid layer thickness), cm. 

### 2.10. UV Photolysis Behavior

To evaluate the photolysis behavior of BPMD and DBPMD under UV irradiation, a photoinitiator solution with a benzophenone group concentration of 5 × 10^−5^ mol/L was prepared using methanol as the solvent. The solution was irradiated using an RX300-1 UV curing machine equipped with a mercury lamp at an intensity of 250 mW/cm^2^ for various durations. The UV absorption spectra of the solution, recorded within the range of 200–350 nm, were analyzed to construct the photoinitiator’s photolysis curve. 

### 2.11. Study of Migration Performance and Extraction Rate

(a) Migration Performance Study: The migration rate of the photoinitiator in HDPE films was assessed by incorporating the photoinitiator and the crosslinker (TAIC) into HDPE (compositions shown in Table 1) and pressing the mixture into 0.1 mm-thick films. The films were irradiated under a mercury lamp at 250 mW/cm^2^ for different durations. Samples (0.1 g) of the irradiated films were immersed in 20 mL of methanol for varying times. The migration behavior was quantified through UV absorption spectra of the methanol solutions. 

(b) Extraction Rate Determination: A control group containing 1% TAIC and 99% HDPE was prepared following the formulation in Table 1. After molding the film, it was irradiated with a mercury lamp at 250 mW/cm^2^ for 30 s. The irradiated film samples (0.1 g) were immersed in 20 mL of methanol for 7 days. The methanol solution’s UV absorption spectrum was used to determine the absorbance at the maximum absorption peak. The extracted photoinitiator amount (m) was calculated using Equation (2):

(2) $$m=M\cdot c\cdot V=\frac{M\cdot A\cdot V}{\varepsilon \cdot l}$$

where m is the amount of extraction; M is the relative molecular weight of the photoinitiator; c is the concentration of photoinitiator, mol/L; V is the volume of methanol solution, L; A is the absorbance at the maximum absorption peak of the photoinitiator; ε is the molar extinction coefficient at the maximum absorption peak of the photoinitiator, L/(mol·cm); l is the optical path length, cm [25].

### 2.12. Initiation Activity

Under UV irradiation, the photoinitiator absorbs UV light, initiating crosslinking by capturing hydrogen atoms from the crosslinker and polyethylene. The activity of the photoinitiator was evaluated by measuring the gel content in UV crosslinked polyethylene film samples.

The gel content was determined as follows: approximately 0.3 g of the crosslinked film was cut into small pieces, weighed (denoted as M_1_) and placed into a stainless steel wire mesh bag with a density of 1000 holes/cm^2^. The weight of the empty mesh bag (denoted as M_2_) and the total weight of the sample and mesh bag (denoted as M_3_) were recorded. The steel wire mesh bag is immersed in a three-mouth flask containing xylene, the three-mouth flask is heated on a heating jacket until xylene is slightly boiled and condensation is maintained, and the mesh bag (denoted as M_4_) is taken out and placed in a 140 °C oven to bake to a constant weight after continuous extraction for 12 h. The gel content was calculated using Equation (3):

(3) $$gel\left(\%\right)=\frac{M_{4}-M_{3}}{M_{2}-M_{1}}\times 100\%$$

### 2.13. Optical Crosslinking Dynamics

The crosslinking process involves the photoinitiator absorbing UV light and initiating crosslinking by capturing hydrogen atoms from the crosslinker and polyethylene. The kinetics of UV induced crosslinking were investigated by varying photoinitiator concentrations, types of crosslinkers and light distances. The gel content of crosslinked polyethylene film samples was measured to analyze the effects of these variables. 

### 2.14. Crystallinity and Mechanical Properties Testing

Crystallinity Measurement: Approximately 6 mg of the sample (accurate to 0.0001 g) was weighed and analyzed using a Q200 DSC instrument from TA Instruments under a nitrogen atmosphere. The temperature was increased from −30 °C to 180 °C at a heating rate of 10 °C/min and the melting endothermic curve was recorded. The melting point was determined from the melting peak. The crystallinity was calculated using Equation (4), based on the melting enthalpy ratio of the sample to that of a 100% crystalline reference [26]:

(4) $$X_{e}=\frac{\Delta H}{\Delta H_{0}}$$

where the 100% crystalline melting enthalpy of polyethylene is 293 J/g. 

Tensile Performance Testing: The tensile properties of sheet samples before and after cross-linking were evaluated following the tensile performance test method outlined in the national standard ISO 527-1:2019 [27]. A universal tensile testing machine (MTS Industrial CMT2103) was employed for the tests. The specific test conditions were as follows: the testing temperature was maintained at 21 °C ± 1 °C with a relative humidity of 63% ± 3%. The width and thickness of the specimen neck were measured with an accuracy of 0.01 mm, and the average of three measurements was recorded. The gauge length was set to 25 mm, and the tensile testing speed was 10 mm/min. For each sample, at least five specimens were tested, and the average value was reported as the final result. 

Creep Performance Testing: The creep resistance of the sheet samples before and after cross-linking modification was assessed according to the creep performance test method specified in ISO 899-1:2017 [28]. The same universal tensile testing machine was utilized for these tests under room temperature and high-stress conditions. The specific test parameters included a temperature of 21 °C ± 1 °C and a relative humidity of 63% ± 3%. Based on the known tensile fracture load of the material, the creep test force was set at 20% of the tensile fracture strength. The fixture length was 25 mm, and the load duration was maintained at 40 min. At least five specimens were tested for each sample, and the average value was reported as the final result. The creep performance of the polyethylene sheets before and after cross-linking was evaluated by analyzing the variation in creep elongation over time (t). 

## 3. Results and Discussion

### 3.1. Characterization of Infrared Absorption Spectroscopy

**Figure 3:**
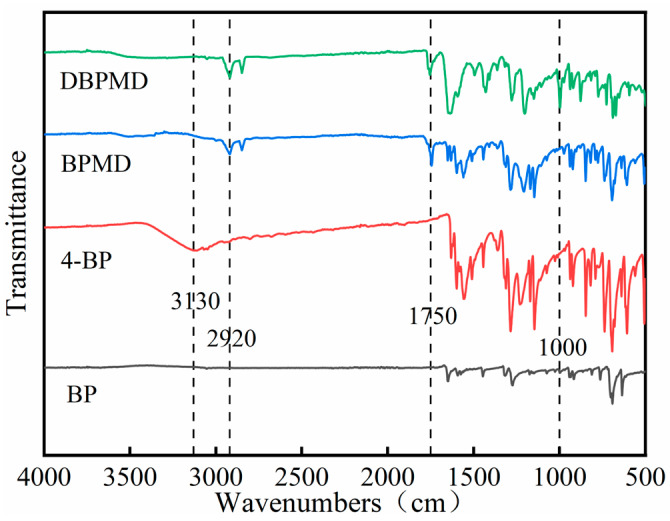
Infrared spectra of BP, 4-BP, BPMD and DBPMD.

Figure 3 illustrates the infrared spectra of BP, 4-BP, BPMD and DBPMD. The phenolic hydroxyl (-OH) characteristic absorption peak at 3130 cm^−1^ in 4-hydroxybenzophenone (4-BP) disappears in the spectra of BPMD and DBPMD. Meanwhile, a new ester (-O-C=O) absorption peak at 1750 cm^−1^ and methylene (-CH_2_-) peaks appear at 2920 cm^−1^ (symmetric stretching vibration) and 2850 cm^−1^ (asymmetric stretching vibration). These changes confirm the reaction between the acyl chloride (-Cl-C=O) group in malonyl chloride and the phenolic hydroxyl group in 4-BP, forming ester groups. 

Additionally, the absorption peak around 1600 cm^−1^ corresponds to the carbonyl (C=O) group intrinsic to 4-BP. The increased methylene peaks in DBPMD compared to BPMD result from the greater number of methylene groups in DBPMD. A distinct carbon-nitrogen (C-N) peak at 1000 cm^−1^ is observed in DBPMD due to the reaction between the acyl chloride group and the secondary amine in piperazine, forming a tertiary amine. In contrast, this peak is absent in BPMD, 4-BP and BP. These results confirm the complete synthesis of BPMD and DBPMD. 

### 3.2. NMR Hydrogen Spectroscopic Characterization

#### 3.2.1. NMR Hydrogen Spectroscopic Characterization of BPMD

**Figure 4:**
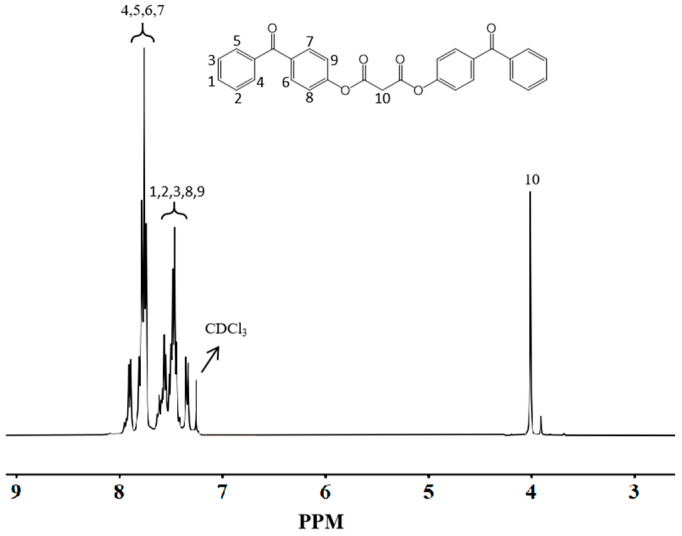
NMR hydrogen spectra of BPMD.

Figure 4 presents the NMR hydrogen spectrum of BPMD. The absorption peaks at δ = 7.89 and δ = 7.70 correspond to the eight hydrogen atoms on the benzene ring adjacent to the carbonyl group in the 4-hydroxybenzophenone structure (positions 4, 5, 6 and 7). Peaks at δ = 7.57, δ = 7.47 and δ = 7.36 are associated with the ten hydrogen atoms on the benzene ring farther from the carbonyl group (positions 1, 2, 3, 8 and 9). 

Additionally, the peak at δ = 4.01 represents the four hydrogen atoms in the methylene groups located between the two acyl groups in the malonyl chloride structure (position 10). The integration of these characteristic peak areas, in conjunction with the expected hydrogen content in the BPMD structure, confirms that the synthesized product is BPMD. 

#### 3.2.2. NMR Hydrogen Spectroscopic Characterization of DBPMD

**Figure 5:**
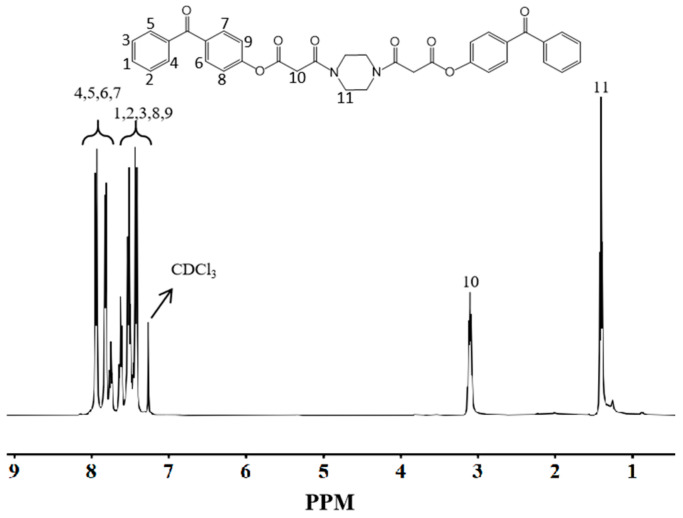
NMR hydrogen spectra of DBPMD.

Figure 5 illustrates the NMR hydrogen spectrum of DBPMD. The absorption peaks at δ = 7.93 and δ = 7.80 represent the eight hydrogen atoms on the benzene ring near the carbonyl group in the 4-hydroxybenzophenone structure (positions 4, 5, 6 and 7). Peaks at δ = 7.62, δ = 7.51 and δ = 7.43 correspond to the ten hydrogen atoms on the benzene ring farther from the carbonyl group (positions 1, 2, 3, 8 and 9). 

The peak at δ = 3.10 reflects the four hydrogen atoms in the methylene group situated between the two acyl groups in the malonyl chloride structure (position 10). Additionally, peaks at δ = 1.40 correspond to the eight hydrogen atoms in the four methylene groups of anhydrous piperazine (position 11). Integration of the characteristic peak areas and comparison with the expected hydrogen content of the DBPMD formula confirm the successful synthesis of DBPMD. 

### 3.3. Mass Spectrometry Characterization

**Figure 6:**
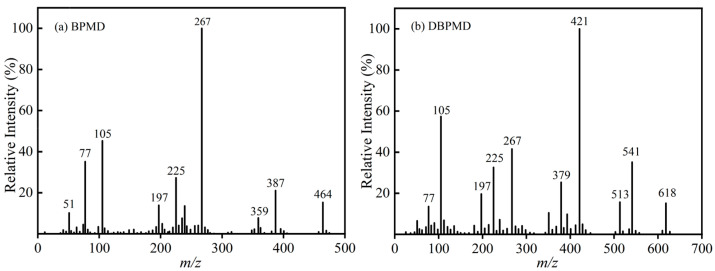
Mass spectrum (**a**) represents BPMD and (**b**) represents DBPMD.

Figure 6a presents the mass spectrum of BPMD, where the molecular ion peak is observed at *m*/*z* = *464*. The peaks at *m*/*z* = *387*, *359*, *105*, and *77* correspond to fragment ions generated by the cleavage of the ketone carbonyl groups on both sides of the benzophenone structure. The peaks at *m*/*z* = *267* and *197* result from the fragmentation of carbon–oxygen bonds within the ester moiety, while the peak at *m*/*z* = *225* arises from the α-cleavage of the ester group. Additionally, the peak at *m*/*z* = *51* is attributed to benzene ring rearrangement.

Figure 6b displays the mass spectrum of DBPMD, with a molecular ion peak at *m*/*z* = *618*. The peaks at *m*/*z* = *541*, *513*, *105*, and *77* correspond to fragment ions formed by the cleavage of the ketone carbonyl groups within the benzophenone structure. The peaks at *m*/*z* = *412*, *225*, and *197* originate from the cleavage of carbonyl groups in the ester moiety, while those at *m*/*z* = *379* and *267* result from fragmentation at additional ketone carbonyl sites.

Analysis of the mass-to-charge ratios and relative peak intensities confirms that the synthesized compounds are BPMD and DBPMD, validating the structural integrity of the target molecules.

### 3.4. Elemental Analysis Characterization

The elemental analysis results of BPMD and DBPMD, obtained using an elemental analyzer, are presented in Table 2. The measured contents of C, H, and O in BPMD were 73.69%, 4.15%, and 22.16%, respectively, with a total deviation of 2.98% from the theoretical values. For DBPMD, the measured contents of C, H, O, and N were 68.32%, 4.63%, 4.39%, and 22.66%, respectively, with a total deviation of 3.94% from the theoretical values. 

### 3.5. DSC Characterization

**Figure 7:**
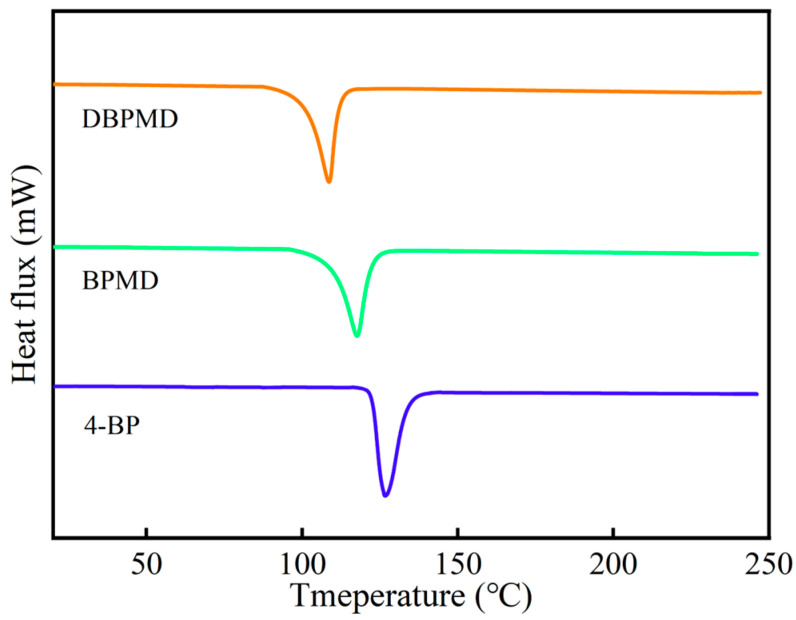
DSC curves of 4-BP, BPMD and DBPMD.

The thermal properties of 4-BP, BPMD and DBPMD were characterized by DSC and the results are shown in Figure 7. 

As shown in Figure 7, the melting points of 4-BP, BPMD, and DBPMD exhibited a gradual decrease, with values of 134.6 °C, 127.3 °C and 119.6 °C, respectively. This reduction in melting point can be attributed to the progressive disruption of the crystal structure as molecular weight increases, leading to a decrease in crystallinity and, consequently, a lower melting point. Given that the typical melting range of HDPE is between 130 °C and 150 °C, a moderately reduced melting point of the photoinitiator can enhance its miscibility with HDPE, facilitating uniform dispersion within the polymer matrix. 

### 3.6. UV Absorption Spectroscopy

**Figure 8:**
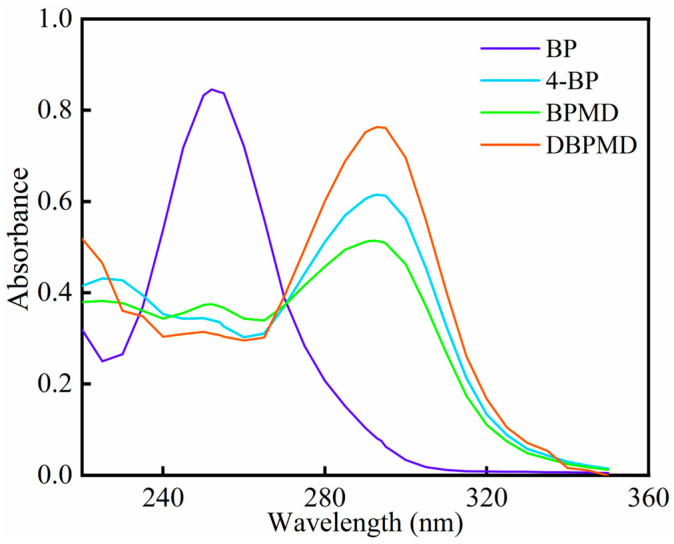
UV absorption spectra of BP, 4-BP, BPMD and DBPMD.

The UV absorption spectra of BP, 4-BP, BPMD and DBPMD were analyzed, and the results are presented in Figure 8 and Table 3. All photoinitiators exhibit UV absorption, with similar absorption behaviors observed among 4-BP, BPMD and DBPMD, except for BP. This indicates that structural modifications do not significantly alter the UV absorption position but increase the maximum absorbance of the photoinitiator. The enhanced absorbance may result from changes in polarity introduced by structural modifications [29]. 

Compared to BP, the maximum absorption wavelength of BPMD and DBPMD shifts from 250 nm to 295 nm, likely due to the substitution of hydroxyl and ester groups on the benzophenone structure [30]. While 4-BP, BPMD and DBPMD exhibit comparable UV absorption behaviors, DBPMD, with double the benzophenone groups of BP and 4-BP, demonstrates a maximum molar extinction coefficient 1.8 times greater than BP and 2.48 times higher than 4-BP. 

### 3.7. UV Photolysis

**Figure 9:**
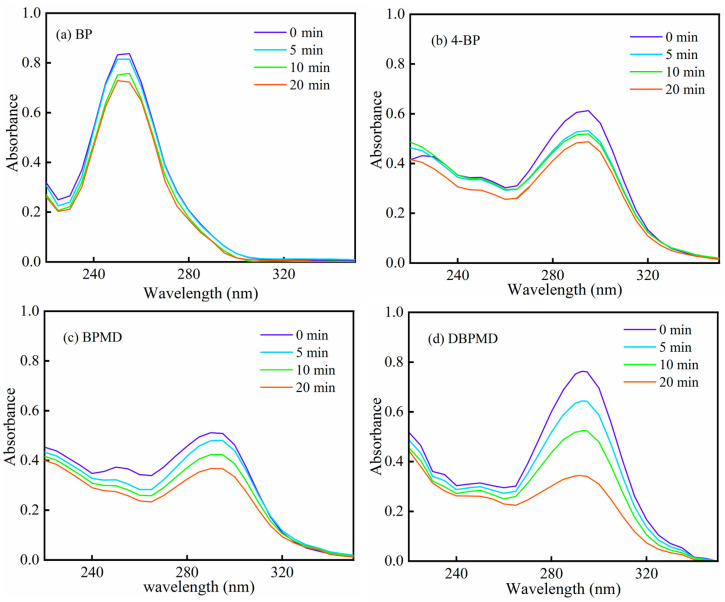
Photolysis curves of each photoinitiator at different times of illumination. (**a**–**d**) represent BP, 4-BP, BPMD and DBPMD, respectively.

Figure 9 presents the photolysis curves of the photoinitiators BP, 4-BP, BPMD and DBPMD under mercury lamp irradiation for varying durations. After 20 min of exposure, the absorbance at the maximum absorption wavelength decreased by 13.66%, 20.40%, 27.92% and 55.06% for BP, 4-BP, BPMD and DBPMD, respectively. The photolysis rates of BPMD and DBPMD exceed those of BP and 4-BP, indicating a more rapid destruction of their conjugated structures, which accelerates the generation of active free radicals to initiate cross-linking.

The faster photolysis of BPMD and DBPMD is attributed to the presence of hydrogen donors, such as tertiary amines, in their structures [31]. Upon irradiation, tertiary amines form excited states Equation (5), enabling hydrogen transfer reactions between excited molecules to produce free radicals Equation (6). This mechanism exhibits greater efficiency compared to smaller molecules like BP and 4-BP. Additionally, the hydroxyl group in 4-BP forms hydrogen bonds with the carbonyl group, reducing its hydrogen capture efficiency and slowing photolysis [32].

R_3_N + *hv* → R_3_N^*^ (5)

where *hv* represents light energy and R_3_N* represents the excited tertiary amine.

R_3_N^*^ + H-R → R_3_N· + R· (6)

where H-R represents the hydrogen donor, R_3_N· represents the tertiary amine radical and R· represents the carbon radical produced by the hydrogen donor after the hydrogen donor is seized. 

### 3.8. Migration Performance

**Figure 10:**
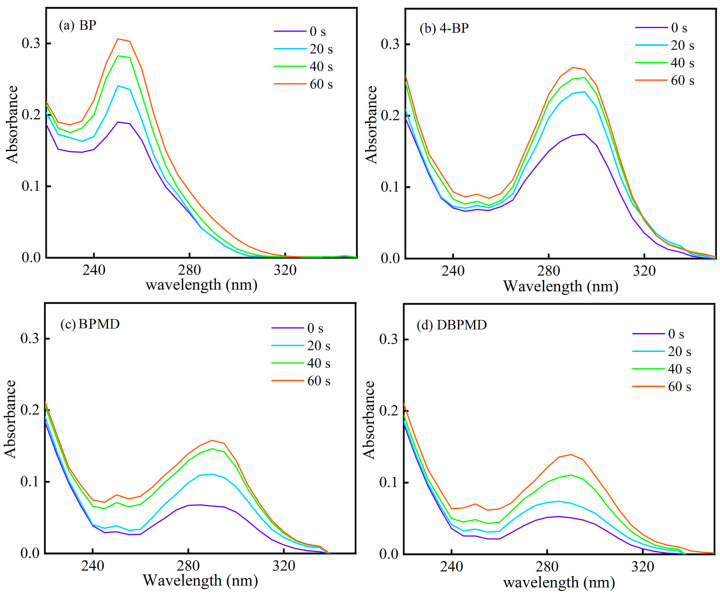
The UV absorption curves of each photoinitiator with UV light time under the same methanol immersion time. (**a**–**d**) represent BP, 4-BP, BPMD and DBPMD respectively.

The migration behavior of BP, 4-BP, BPMD and DBPMD was evaluated by analyzing changes in their UV absorption curves under varying UV irradiation times and methanol immersion durations. Figure 10 illustrates that the absorbance of each photoinitiator system increased with prolonged UV exposure, with the growth rate plateauing after 40 s of irradiation. Compared to BP, 4-BP and BPMD, the maximum absorbance wavelength of DBPMD exhibited a respective decrease of 54.60%, 47.43% and 9.38%, indicating its superior migration resistance under the same conditions. 

**Figure 11:**
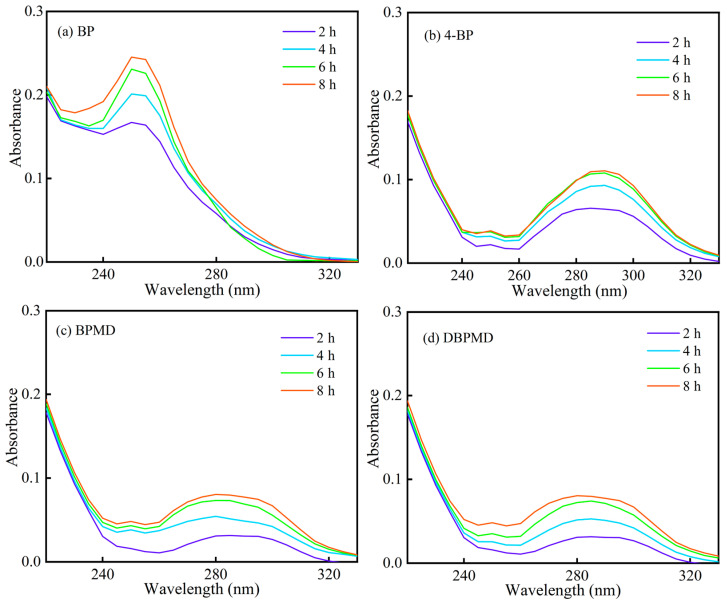
The UV absorption curves of each photoinitiator with methanol immersion time under the same UV illumination time. (**a**–**d**) represent BP, 4-BP, BPMD and DBPMD, respectively.

As shown in Figure 11, the absorbance of all systems increased with extended methanol immersion time, plateauing after 6 h. After 8 h of immersion, the absorbance at the maximum absorption wavelength of DBPMD decreased by 67.57%, 27.87% and 1.00% compared to BP, 4-BP and BPMD, respectively. These results indicate that DBPMD exhibits the lowest absorbance and slowest growth rate, followed by BPMD, suggesting their limited migration within polyethylene. This reduced migration is attributed to the large molecular weight and elongated structures of BPMD and DBPMD, which are constrained within the polyethylene crosslinking network. Furthermore, macromolecular photoinitiators like BPMD and DBPMD stabilize themselves within the network through intra- or intermolecular hydrogen-capture cross-linking, effectively reducing self-migration and extraction rates [33].

### 3.9. Determination of Extraction Amount

**Figure 12:**
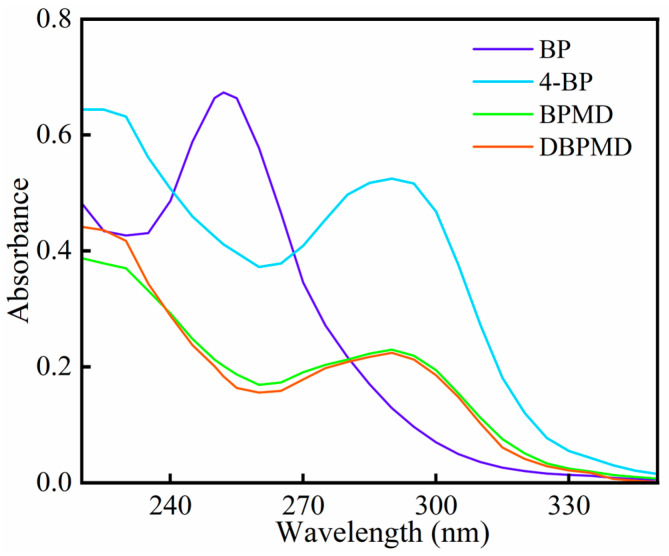
UV absorption of polyethylene membranes of each system after soaking for 7 days.

Figure 12 illustrates the UV absorption spectra of crosslinked polyethylene films containing BP, 4-BP, BPMD and DBPMD after being soaked in 20 mL of methanol for 7 days. The data reveal that a minor quantity of photoinitiator is still leached from the crosslinked polyethylene films. By applying Equation (2), the extraction amount of each photoinitiator was calculated based on the absorbance at the maximum absorption wavelength. The extraction amounts, expressed as percentages of the initial photoinitiator quantities, are summarized in Table 4. 

After soaking in methanol for 7 days, the extraction rates of polyethylene membranes for the photoinitiators BP, 4-BP, BPMD and DBPMD were determined to be 14.7%, 16.9%, 10.1% and 8.7%, respectively. The lower extraction rates of the macromolecular photoinitiators, BPMD and DBPMD, can be attributed to two primary factors: their higher molecular weights, which restrict movement and migration within polyethylene and the high crosslinking degree of the system, which entraps photoinitiators in the three-dimensional crosslinking network, thereby reducing mobility and extractability [34]. 

### 3.10. Eliciting Activity Contrasts

**Figure 13:**
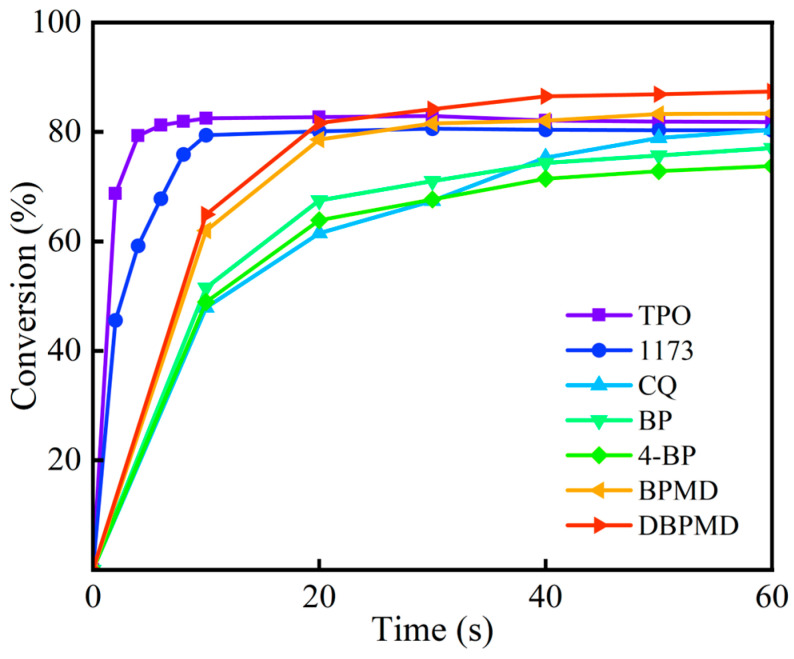
The gel content of each system varies with light time.

To compare the gel content of BP, 4-BP, BPMD, and DBPMD with commonly used photoinitiators TPO, 1173, and CQ after mixed crosslinking with the crosslinker TAIC, seven photocrosslinking systems were prepared. Each system contained one of the photoinitiators (TPO, 1173, CQ, BP, 4-BP, BPMD, or DBPMD) along with TAIC to initiate polyethylene crosslinking under UV irradiation at an intensity of 250 mW/cm^2^. Figure 13 presents the gel content variation as a function of irradiation time for the seven systems. For the systems containing TPO and 1173, gel content measurements were taken every 2 s during the first 10 s of irradiation and subsequently at 10-s intervals. For the other systems, gel content was measured every 10 s up to 60 s. 

As shown in Figure 13, TPO exhibited a rapid initiation rate, achieving a gel content of 79.32% within the first 4 s, which increased marginally to 81.83% after 60 s of UV exposure. This result indicates that TPO facilitates cross-linking at a significantly high rate. In comparison, 1173 reached a gel content of 79.45% within approximately 10 s, with a final value of 80.28% at 60 s, demonstrating a slightly slower cross-linking rate than TPO. The initiation rate of CQ was similar to that of BP, with gel contents of 80.41%, 77.05%, 73.76%, 83.35% and 87.39% for CQ, BP, 4-BP, BPMD, and DBPMD, respectively. Notably, BPMD and DBPMD exhibited significantly higher gel content than BP and 4-BP. This enhancement can be attributed to the higher molecular weight and longer molecular chains of BPMD, which reduce its migration to the film surface during the mixing and pressing process. Consequently, BPMD tends to exhibit localized and uneven distribution, leading to either limited cross-linking or surface cross-linking only [25]. In contrast, DBPMD not only possesses a higher molecular weight and extended molecular chains but also incorporates an amine hydrogen donor within its molecular structure [35]. This feature allows DBPMD to rapidly initiate matrix cross-linking by generating amino radicals via intermolecular hydrogen abstraction. TPO and 1173 are class I photoinitiators, characterized by fast initiation kinetics, whereas CQ and BP belong to class II photoinitiators, which require amine-based co-initiators to enhance their initiation efficiency due to their inherently slower initiation rates. While BP photoinitiators are more cost-effective compared to TPO and 1173, they require active amine additives to improve their initiation efficiency. However, a key drawback of BP-based photoinitiators is their tendency to induce severe yellowing in HDPE upon prolonged UV exposure. The cross-linker plays a crucial role in facilitating UV-induced cross-linking initiated by the photoinitiator. During the process, the photoinitiator abstracts hydrogen atoms from both polyethylene and the cross-linker, generating corresponding free radicals. These radicals enable polyethylene and cross-linker molecules to interact, forming interconnected polyethylene macromolecular chains. The resulting cross-linked structures arise through bimolecular coupling and disproportionation termination reactions, as illustrated in Equations (7) and (8).

PE· + TAIC· → PE-TAIC-PE (7)

In the bi-group coupling reaction, in which PE· is a polyethylene radical and TAIC· is a free radical of the cross-linking agent TAIC, the cross-linked structure generated by the reaction connects the different polyethylene chains through the cross-linker.

TAIC· + PE· → TAIC-H + PE = CH_2_ (8)

The bibase disproportionation termination reaction is that the free radicals of the crosslinker disproportionate with the polyethylene chain free radicals and the chain growth is terminated. 

### 3.11. Optical Cross-Linking Dynamics Studies

#### 3.11.1. Effect of Photoinitiator Concentration on Photocrosslinking Kinetics

**Figure 14:**
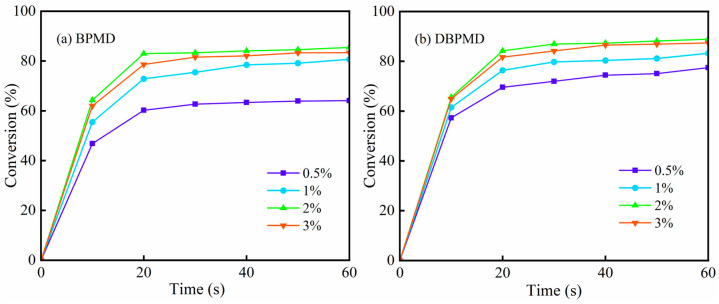
Effect of photoinitiator concentration on gel content. (**a**) represents BPMD and (**b**) represents DBPMD.

To investigate the effect of photoinitiator concentration on the kinetics of photocrosslinking, eight systems were prepared with BPMD and DBPMD at mass fractions of 0.5%, 1%, 2% and 3%, respectively, and a fixed 1% mass fraction of the crosslinker TAIC. Polyethylene crosslinking was initiated under UV light with an intensity of 250 mW/cm^2^. As shown in Figure 14, the gel contents of BPMD systems were 64.09%, 80.66%, 85.46% and 83.35%, while those of DBPMD systems were 77.45%, 83.23%, 88.87% and 87.39%, respectively. Gel content increased with photoinitiator concentration from 0.5% to 2%, but declined slightly at 3%. This reduction may result from excessive local excitation of the photoinitiator, leading to self-quenching and a decrease in the photoinitiation rate. Therefore, optimizing the photoinitiator concentration is crucial to enhance the efficiency of crosslinking in polyethylene systems.

#### 3.11.2. Effect of Crosslinker Type on Optical Crosslinking Kinetics

**Figure 15:**
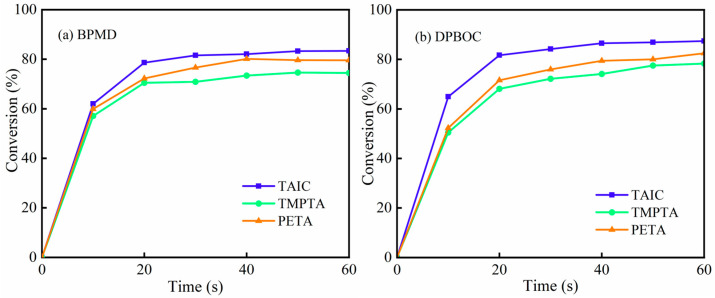
Effect of crosslinker type on gel content. (**a**) represents BPMD and (**b**) represents DBPMD.

In order to study the influence of crosslinking agent types on photocrosslinking kinetics, six systems were prepared using BPMD and DBPMD at a fixed 1% mass fraction, combined with the crosslinkers TAIC, TMPTA and PETA, and exposed to 1% light intensity to initiate polyethylene crosslinking. As shown in Figure 15, the final gel contents of BPMD and DBPMD systems were 83.35%, 74.44%, 79.56%, 87.39%, 78.30% and 82.45%, respectively. The crosslinking efficiency of TAIC was superior to that of TMPTA and PETA, attributed to the triazine ring in TAIC, which enhances thermal stability and penetration depth. 

#### 3.11.3. Effect of Light Distance on Optical Crosslinking Dynamics

**Figure 16:**
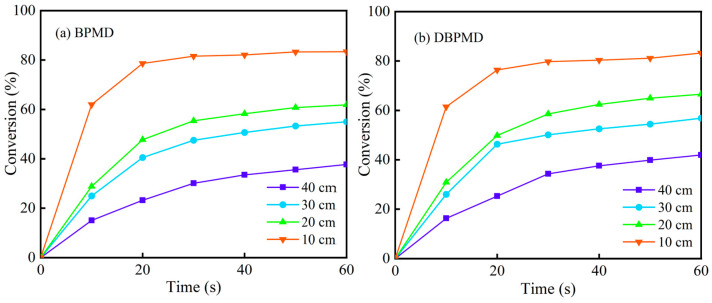
Variation of gel content with light time at different light distances. (**a**) represents BPMD and (**b**) represents DBPMD.

To investigate the effect of light distance on the kinetics of photocrosslinking, two systems were prepared using the photoinitiators BPMD and DBPMD, along with 1% of the crosslinker TAIC. The experiments were conducted under a fixed light intensity of 250 mW/cm^2^, with illumination distances of 10 cm, 20 cm, 30 cm and 40 cm, respectively. As shown in Figure 16, the final gel contents for BPMD were 37.71%, 55.02%, 61.87% and 83.35%, while for DBPMD, they were 41.49%, 56.86%, 66.56% and 87.39%. An increase in light distance resulted in a reduction in gel content and a longer light exposure time required to reach the maximum gel content. This can be attributed to the reduction in light intensity and UV energy absorption as the light distance increases, leading to a slower formation of the excited state of the photoinitiator.

### 3.12. Crystallinity and Mechanical Properties

To investigate the relationship between the crystallinity of cross-linked polyethylene and the concentration of the photoinitiator, two systems with different concentrations of photoinitiators, BPMD and DBPMD, were prepared. DSC analysis was performed after 30 s of UV irradiation at an intensity of 250 mW/cm^2^. Additionally, to determine whether polyethylene crosslinking primarily occurs in the crystalline or amorphous regions, the gel content of the cross-linked polyethylene film was measured. The polyethylene retained in the cross-linked portion within the mesh bag and the polyethylene dissolved in the uncrosslinked fraction extracted by xylene were analyzed using DSC. Furthermore, to evaluate the stress, strain, and creep behavior of polyethylene cross-linked by different photoinitiators, four systems containing 1% photoinitiator and a cross-linking agent were prepared, along with a blank system without a photoinitiator. Dumbbell-shaped samples with a thickness of 0.1 mm were prepared and tested after 30 s of UV irradiation. 

#### 3.12.1. Crystallinity Studies

**Figure 17:**
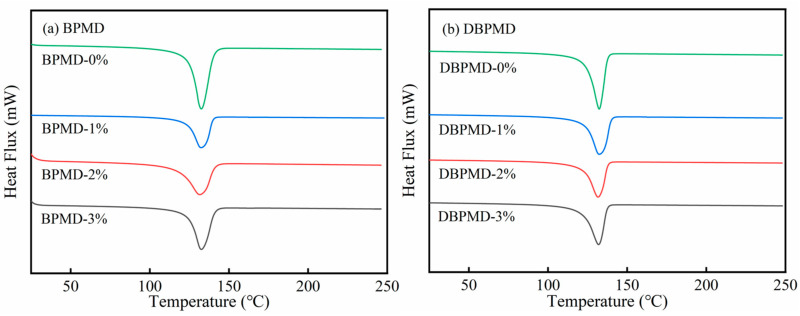
DSC with different photoinitiation dose systems. (**a**) represents BPMD and (**b**) represents DBPMD.

From the data presented in Figure 17 and Table 5, it is evident that increasing the photoinitiator content slightly reduces the melting point, though the shift is minimal. When the photoinitiator BPMD and DBPMD concentrations increased from 0% to 1%, crystallinity decreased significantly by 6.97% and 6.63%, respectively. However, as the photoinitiator content continued to rise, crystallinity showed a slight recovery but remained noticeably lower than in systems without photoinitiator. This phenomenon may be attributed to cross-linking disrupting parts of the crystal structure, while limited new crystal structures form as cross-linking progresses, leading to a marginal crystallinity increase [36]. 

**Figure 18:**
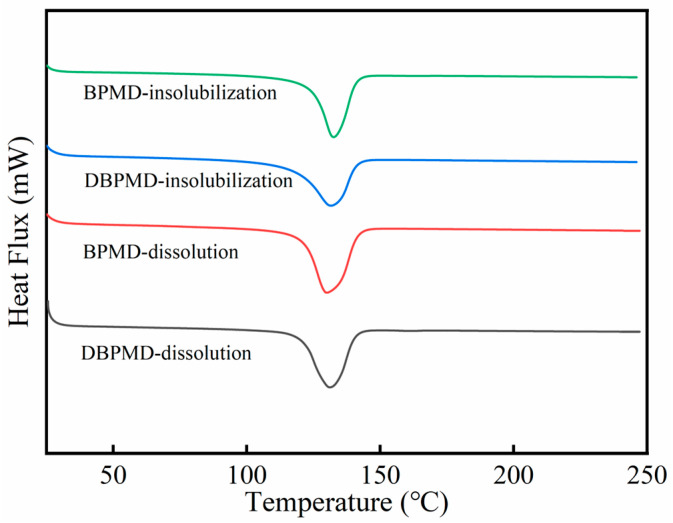
Polyethylene DSC in the undissolved and dissolved fractions.

As illustrated by the data in Figure 18 and Table 5, the crystallinity of the undissolved cross-linked portion is lower than that of the uncross-linked portion dissolved in xylene, indicating that polyethylene cross-linking predominantly occurs in the amorphous regions. This is because molecular chains in the crystalline regions are tightly organized into lattice structures with strong mechanical interactions, making cross-linking difficult [37]. In contrast, polymer chains in the amorphous regions are randomly entangled and capable of sliding relative to each other, facilitating cross-linking reactions. 

#### 3.12.2. Tensile Properties Study

**Figure 19:**
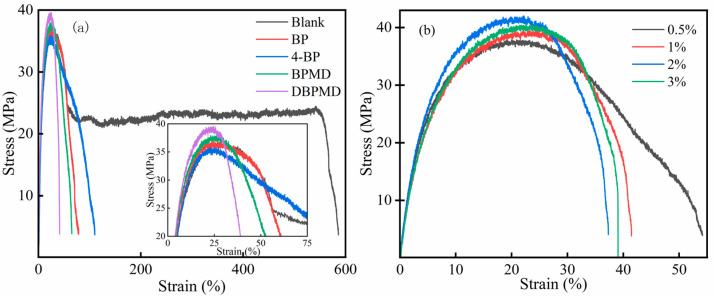
Stress-strain curves of each system after light crosslinking. (**a**) represents the stress-strain curves after photocrosslinking of each system, and (**b**) represents the stress-strain curves after photocrosslinking of DBPMD at different concentrations.

Figure 19 illustrates the stress-strain curves for the five systems (a) and the DBPMD system (b), while Table 6 summarizes the tensile yield strength and elongation at break for these systems at varying DBPMD concentrations. The data indicate that the addition of photoinitiators slightly increases tensile strength but significantly reduces elongation at break. For specimens without photoinitiators, stress induces yield and cold drawing, forming a thin neck; stress then increases with strain until strain hardening and eventual fracture. Conversely, photoinitiator-containing specimens exhibit immediate fracture after yielding, with minimal elongation at break [38]. 

As the DBPMD concentration in polyethylene increases, the degree of cross-linking rises, leading to a slight increase in tensile strength and a notable decrease in elongation at break. However, excessive DBPMD concentrations reduce cross-linking, causing a decrease in tensile strength and an increase in elongation at break. These observations suggest that cross-linking forms a three-dimensional network structure, which severely entangles molecular chains, reducing chain orientation during stretching. Additionally, cross-linked structures introduce entanglement points within polyethylene, which concentrate stress, slightly reducing tensile strength and transitioning the material from ductile to brittle behavior [39,40]. 

#### 3.12.3. Creep Performance Studies

**Figure 20:**
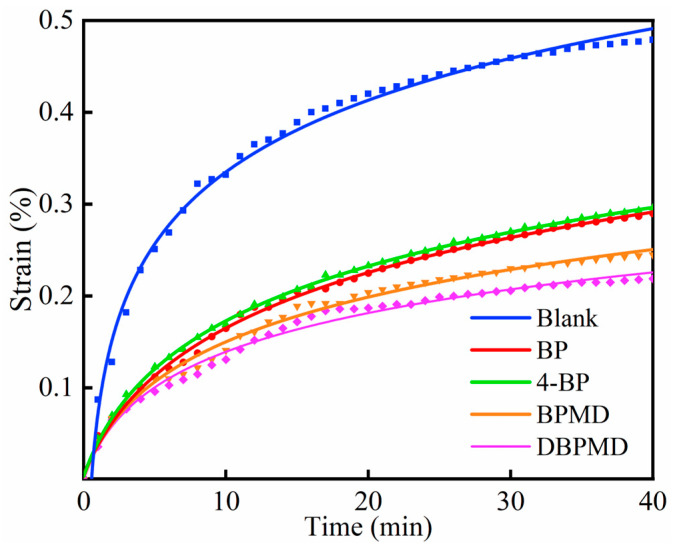
Creep curves of each system after light cross-linking.

Figure 20 illustrates the creep elongation of five systems over time. Both cross-linked and non-cross-linked samples exhibit an initial rapid increase in creep elongation, which subsequently slows and stabilizes. However, the creep elongation rate of the non-cross-linked blank sample is significantly higher than that of the cross-linked samples. Among the cross-linked samples, those with BPMD and DBPMD demonstrate lower creep elongation compared to BP and 4-BP, attributed to their higher cross-linking degree. The formation of a three-dimensional network structure in these systems entangles molecular chains, restricts chain slippage and reduces creep elongation [40,41]. 

After 40 min under tensile force, the creep elongation of BP, 4-BP, BPMD and DBPMD systems decreased by 39.45%, 38.20%, 48.85% and 54.28%, respectively, compared to the non-photoinitiator blank system. These results confirm that higher cross-linking effectively limits molecular movement, enhancing dimensional stability under stress. 

## 4. Conclusions

Two macromolecular photoinitiators, BPMD and DBPMD, were synthesized, with DBPMD incorporating an amine-based hydrogen donor. (a) UV spectroscopy and gel content analysis revealed that the molar extinction coefficient, photolysis rate and gel content of BPMD and DBPMD systems exceeded those of BP and 4-BP systems, and the maximum absorbance wavelengths of BPMD and DBPMD showed a red shift compared to BP. (b) UV absorption spectroscopy indicated that the migration rates of BPMD and DBPMD in cross-linked polyethylene films were significantly lower than those of BP and 4-BP. (c) Mechanical testing demonstrated that polyethylene films cross-linked with BPMD and DBPMD exhibited superior tensile strength and creep resistance compared to those cross-linked with BP and 4-BP. 

## Figures and Tables

**Table 1:** Formulation of photoinitiators, cross-linking agents, and HDPE (w%).

	BP	4-BP	BPMD	DBPMD	TAIC	HDPE
a	1				1	98
b		1			1	98
c			1		1	98
d				1	1	98

**Table 2:** Elemental analysis of BPMD and DBPMD.

Photoinitiators	Element	Measured Value (%)	Theoretical Value (J/g)	Deviation (%)
BPMD	C	73.69	74.99	−1.30
	H	4.15	4.34	−0.19
	N	0.00	0.00	0.00
	O	22.16	20.67	+1.49
DBPMD	C	68.32	69.89	−1.57
	H	4.63	4.89	−0.26
	N	4.39	4.53	−0.14
	O	22.66	20.69	+1.97

**Table 3:** Light absorbance data of BP, 4-BP, BPMD and DBPMD.

Photoinitiators	λ_max_/nm	ε_max_/(L·mol^−1^·cm^−1^)
BP	252	16,912
BP	283	1510
4-BP	283	12,292
BPMD	283	20,568
DBPMD	283	30,528

**Table 4:** Extraction rates of BP, 4-BP, BPMD, and DBPMD.

Photoinitiators	A_max_	Extraction Quality 10^−4^ (g)	Extraction Rate (%)
BP	0.6734	1.45	14.5
4-BP	0.5248	1.69	16.9
BPMD	0.2298	1.01	10.1
DBPMD	0.2244	0.87	8.7

**Table 5:** Crystallinity of BPMD and DBPMD systems.

Photoinitiators	Quality Score (w%)	Melting Point (°C)	Enthalpy of Fusion (J/g)	Crystallinity (%)
BPMD	0	132.62	224.88	76.75
	1	132.03	204.37	69.78
	2	131.46	206.33	70.42
	3	131.13	208.70	71.23
DBPMD	0	132.62	224.88	76.75
	1	131.98	205.45	70.12
	2	131.35	207.71	70.89
	3	131.05	209.79	71.56

**Table 6:** Mechanical properties of each system after light cross-linking.

Photoinitiators	Quality Score (w%)	Tensile Strength (MPa)	Elongation at Break (%)
-	-	36.62	586
BP	1	35.81	79
4-BP	1	35.31	110
BPMD	1	37.77	65
DBPMD	1	39.34	41
DBPMD	0.5	37.61	51
DBPMD	2	40.82	37
DBPMD	3	39.59	39

## Data Availability

The data that support the findings of this study are available from the corresponding author upon reasonable request.

## References

[1] Selvin M., Shah S., Maria H.J., Thomas S., Tuladhar R., Jacob M. (2024). Review on recycling of cross-linked polyethylene. Ind. Eng. Chem. Res..

[2] Kouhi M., Butan S., Li Y., Shakour E., Banu M. (2021). Role of chemically functionalization of bamboo fibers on polyethylene-based composite performance: A solution for recycling. Polymers.

[3] Pearson A., Duncan M., Hammami A., Naguib H.E. (2022). Interfacial adhesion and thermal stability of high-density polyethylene glass fiber composites. Compos. Sci. Technol..

[4] Mao Q., Su B., Ma R., Li Z. (2021). Investigation of Tensile Creep Behavior for High-Density Polyethylene (HDPE) via Experiments and Mathematical Model. Materials.

[5] Wang F., Yu J., Liu L., Xue P., Chen K. (2023). Influence of high-density polyethylene content on the rheology, crystal structure, and mechanical properties of melt spun ultra-high-molecular weight polyethylene/high-density polyethylene blend fibers. J. Ind. Text..

[6] Zha S., Lan H.-Q., Huang H. (2022). Review on lifetime predictions of polyethylene pipes: Limitations and trends. Int. J. Press. Vessel. Pip..

[7] Wang H., Xu L., Li R., Hu J., Wang M., Wu G. (2016). Improving the creep resistance and tensile property of UHMWPE sheet by radiation cross-linking and annealing. Radiat. Phys. Chem..

[8] Luo Y., Zhou T., Jin T., Wang C., Ma Y., Song H., Zhang H. (2025). Study on the low-temperature performance of chemically cross-linked polyethylene composite modified asphalt binder. Chem. Eng. Sci..

[9] Zhang J., Li J., Li H., Yang M., Li Y., Qi Y., Xu Y., Hu W., Liu B. (2024). Recent Overview and Future Research Prospects of Cross-linked Polyethylene Materials: Cross-linking Methods and Applications. Preprints.

[10] Chodák I. (1998). High modulus polyethylene fibres: Preparation, properties and modification by crosslinking. Prog. Polym. Sci..

[11] Yen S.C., Ni J.S., Chen Y.C. (2024). Synthesis of One-Component Type II Visible-Light-Absorbing Chalcones Containing Fused Aromatic Rings and Investigation of Free Radical Photopolymerization Properties. Macromol. Chem. Phys..

[12] Dietliker K., Hüsler R., Birbaum J.-L., Ilg S., Villeneuve S., Studer K., Jung T., Benkhoff J., Kura H., Matsumoto A. (2007). Advancements in photoinitiators—Opening up new applications for radiation curing. Prog. Org. Coat..

[13] Zhang H., Shang Y., Li M., Zhao H., Wang X., Han B. (2016). Theoretical study on the reaction mechanism in the UV radiation cross-linking process of polyethylene. RSC Adv..

[14] Wang X., Li W., Nie J., Zhu X. (2024). A deep-curing UV-LED light photoinitiator based on diphenylpropanetrione. J. Photochem. Photobiol. A Chem..

[15] Duan K., Chen X., Ni J., Mei Z., Zhou C., Ye X., Zhu H., Yu D. (2024). Preparation and properties of ultraviolet crosslinked high-density polyethylene materials. China Plast..

[16] Dong T., Niu F., Qiang Z., Wang X., Guo J., Wang Y., He Y. (2024). Structure and properties evolution of UV crosslinked ultra-high molecular weight polyethylene fiber during processing. J. Polym. Sci..

[17] Cınar S.A., Guven M.N., Eren T.N., Cesur B., Aleksanyan M., Dedeoglu B., Okte N., Aviyente V., Morlet-Savary F., Lalevée J. (2016). Structure-reactivity relationships of novel monomeric photoinitiators. J. Photochem. Photobiol. A Chem..

[18] Cesur B., Karahan O., Agopcan S., Eren T.N., Okte N., Avci D. (2015). Difunctional monomeric and polymeric photoinitiators: Synthesis and photoinitiating behaviors. Prog. Org. Coat..

[19] Li W., Nie J., Zhao Y., Zhu X. (2024). Photoinitiators with low migration capability based on benzophenone. Eur. Polym. J..

[20] Zhou J., Allonas X., Ibrahim A., Liu X. (2019). Progress in the development of polymeric and multifunctional photoinitiators. Prog. Polym. Sci..

[21] Taschner R., Gauss P., Knaack P., Liska R. (2020). Biocompatible photoinitiators based on poly-α-ketoesters. J. Polym. Sci..

[22] Davidson R.S., Dias A.A., Illsley D. (1995). A new series of type II (benzophenone) polymeric photoinitiators. J. Photochem. Photobiol. A Chem..

[23] Huang T.-L., Chen Y.-C. (2022). Synthesis and free radical photopolymerization of one-component type II photoinitiator based on benzophenone segment. J. Photochem. Photobiol. A Chem..

[24] Li W., Nie J., Zhao Y., Zhu X. (2024). A low mobility UV-LED benzophenone photoinitiator. J. Photochem. Photobiol. A Chem..

[25] Deng L., Tang L., Qu J. (2020). Synthesis and photopolymerization of novel UV-curable macro-photoinitiators. Prog. Org. Coat..

[26] Huang T.-L., Chen Y.-C. (2021). Ketone number and substitution effect of benzophenone derivatives on the free radical photopolymerization of visible-light type-II photoinitiators. Polymers.

[27] Paradowska-Stolarz A., Malysa A., Mikulewicz M. (2022). Comparison of the compression and tensile modulus of two chosen resins used in dentistry for 3D printing. Materials.

[28] Jasem M.H., Abbod E.A., Jassim T.A., Hussien Z.Y., Omaraa E. (2025). Steady-State Creep Behaviour of Functionally Graded Silicone Rubber with Cellulose Addition. J. Eng. Sustain. Dev..

[29] Cheng L., Shi W. (2011). Synthesis and photoinitiating behavior of benzophenone-based polymeric photoinitiators used for UV curing coatings. Prog. Org. Coat..

[30] Feng C., Wang Q.L., Liu F., Zhang B. (2023). Synthesis and application of new benzophenone photoinitiators. ChemistrySelect.

[31] Dumur F. (2022). Recent advances on visible light Triphenylamine-based photoinitiators of polymerization. Eur. Polym. J..

[32] Davidson R.S., Dias A.A., Illsley D.R. (1995). Type II polymeric photoinitiators (polyetherimides) with built-in amine synergist. J. Photochem. Photobiol. A Chem..

[33] Deka S., Kakati D. (2009). Benzoin-terminated polyurethane as macrophotoinitiator for synthesis of polyurethane–polymethyl methacrylate block copolymers. J. Appl. Polym. Sci..

[34] Liang Q., Wang Z., Du W., Liu W., Cao J., Ren J., Lian W., Lu H., Li H. (2022). Determination of 18 photoinitiators in food paper packaging materials by FastPrep-based extraction combined with GC–MS. Food Chem..

[35] Sanai Y., Ninomiya T., Arimitsu K. (2021). Improvements in the physical properties of UV-curable coating by utilizing type II photoinitiator. Prog. Org. Coat..

[36] Zhang Q., Zhu G.-R., Xiao X.-X., Liu Q.-S., Jiang M., Guo D.-M., Zhao H.-B., Li W.-D., Chen L., Liu B.-W. (2023). Controllable micro cross-linking towards multifunctional flame-retardant aliphatic polyamide. Chem. Eng. J..

[37] Cho H.-K., Kim H. (2024). Preparation of de-crosslinked polyethylene from waste crosslinked high-density polyethylene using supercritical twin-screw extrusion. Korean J. Chem. Eng..

[38] Bobovitch A., Gutman E.M., Henning S., Michler G.H. (2003). Morphology and stress-relaxation of biaxially oriented cross-linked polyethylene films. Mater. Lett..

[39] Datta S., Stocek R., Naskar K. (2021). Influence of ultraviolet radiation on mechanical properties of a photoinitiator compounded high vinyl styrene–butadiene–styrene block copolymer. Polymers.

[40] Montoya-Ospina M.C., Verhoogt H., Ordner M., Tan X., Osswald T.A. (2022). Effect of cross-linking on the mechanical properties, degree of crystallinity and thermal stability of polyethylene vitrimers. Polym. Eng. Sci..

[41] Mao H.-D., Zhang T.-T., Guo Z.-Y., Bai D.-Y., Wang J., Xiu H., Fu Q. (2023). A cross-linked polyethylene with recyclability and mechanical robustness enabled by establishment of multiple hydrogen bonds network via reactive melt blending. Chin. J. Polym. Sci..

